# Relationships of Cannabis Policy Liberalization With Alcohol Use and Co-Use With Cannabis: A Narrative Review

**DOI:** 10.35946/arcr.v42.1.06

**Published:** 2022-03-17

**Authors:** Rosalie Liccardo Pacula, Rosanna Smart, Marlene C. Lira, Seema Choksy Pessar, Jason G. Blanchette, Timothy S. Naimi

**Affiliations:** 1 University of Southern California, Los Angeles, California, USA; 2 RAND Corporation, Santa Monica, California, USA; 3 Boston Medical Center, Boston, Massachusetts, USA; 4 Boston University School of Public Health, Boston, Massachusetts, USA; 5 Canadian Institute for Substance Use Research, Victoria, British Columbia, Canada

**Keywords:** cannabis, marijuana, policy, alcohol, outcomes, co-use, public health

## Abstract

**PURPOSE:**

The liberalization of cannabis policies has the potential to affect the use of other substances and the harms from using them, particularly alcohol. Although a previous review of this literature found conflicting results regarding the relationship between cannabis policy and alcohol-related outcomes, cannabis policies have continued to evolve rapidly in the years since that review.

**SEARCH METHODS:**

The authors conducted a narrative review of studies published between January 1, 2015, and December 31, 2020, that assessed the effects of cannabis policies on the use of alcohol in the United States or Canada.

**SEARCH RESULTS:**

The initial search identified 3,446 unique monographs. Of these, 23 met all inclusion criteria and were included in the review, and five captured simultaneous or concurrent use of alcohol and cannabis.

**DISCUSSION AND CONCLUSIONS:**

Associations between cannabis policy liberalization and alcohol use, alcohol-related outcomes, and the co-use of alcohol and cannabis were inconclusive, with studies finding positive associations, no associations, and negative associations. Although several studies found that cannabis policy liberalization was associated with decreases in alcohol use measures, these same studies showed no impact of the cannabis policy on cannabis use itself. The lack of a consistent association was robust to subject age, outcome measure (e.g., use, medical utilization, driving), and type of cannabis policy; however, this may be due to the small number of studies for each type of outcome. This paper discusses several notable limitations of the evidence base and offers suggestions for improving consistency and comparability of research going forward, including a stronger classification of cannabis policy, inclusion of measures of the alcohol policy environment, verification of the impact of cannabis policy on cannabis use, and consideration of mediation effects.

For the past 25 years, a growing number of U.S. states have been progressively legalizing cannabis markets, first through the early adoption of medical cannabis laws, which enabled the purchase and possession of cannabis for specific medicinal purposes, and then more recently through laws regarding adult (i.e., “recreational”) use of cannabis. As of May 2021, more than 70% of U.S. states (*n* = 36) allowed for medical markets of cannabis and 18 states and the District of Columbia had passed laws allowing for the recreational use of cannabis by adults,^1^ despite federal prohibition. A key public health concern throughout much of this state policy innovation over the past 2 decades is the impact these cannabis liberalization laws might have on alcohol use and alcohol-related harm.^2^,^3^ Although the harms caused by persistent use of cannabis, particularly high-potency cannabis, are still under scientific investigation,^4^,^5^ the known harms associated with alcohol use are well established.^6^

Some have argued that cannabis use may be a substitute for alcohol consumption and, therefore, that liberalizing cannabis policies should reduce excessive drinking and alcohol-related harms. However, during the past 2 decades, there has been a consistent upward trend in alcohol consumption, as measured by per capita consumption^7^,^8^ and self-reported annual and 30-day alcohol use prevalence rates.^9^ This has occurred during the same period as a liberalization of cannabis policies. Significant research shows that cannabis use is a risk factor for underage drinking, excessive drinking, and crash fatalities involving alcohol,^10^ supporting the notion that the liberalizing of cannabis policies may be contributing to the rise in alcohol use. Cannabis use among people who report drinking in the past month or past year (i.e., concurrent use among drinkers) remains fairly low overall, with approximately 15% of drinkers reporting cannabis use in the same month or past year.^11^–^13^ However, concurrent use of alcohol among cannabis users is quite a bit higher, with more than 75% of cannabis users reporting alcohol use within the same 30-day period.^14^–^17^ As more liberal cannabis laws generate more adult cannabis users,^18^ there is concern that such laws may be resulting in more concurrent use of cannabis and alcohol as well.

Even more disconcerting is the evidence that two-thirds of those who both drink alcohol and use cannabis consume the substances simultaneously^11^–^13^—that is, during the same occasion. Recent evidence from the Monitoring the Future survey shows that the prevalence of simultaneous use of alcohol and marijuana (SAM) among young adults who drink (ages 19 to 22) is as high as 30%, and that of slightly older young adults (ages 23 to 30) is between 20% and 25%.^19^ Moreover, between 1992 and 2016, there has been a consistent and significant increase in the prevalence of SAM among people ages 21 to 26 who drink alcohol, although the prevalence of SAM among people who use cannabis has been relatively stable.^19^

This growing evidence of simultaneous use of these two substances among people who drink alcohol has some public health advocates concerned that cannabis liberalization policies may be leading to more, not less, alcohol use and even more concurrent or simultaneous use. Compared with alcohol use alone, studies have shown two to three times increased odds of adverse social consequences (e.g., legal issues, relationship and financial problems) associated with co-use of alcohol and cannabis,^12^,^13^,^20^ and simultaneous use is known to lead to greater cognitive, perceptual, and motor function impairment than using either alcohol or cannabis alone.^4^,^21^–^23^

The relationship between the consumption of cannabis and alcohol in a population will be influenced by a multitude of individual and environmental factors, including socio-demographics, cultural norms, perceived harm, and the general availability of both substances. Policy plays a role in influencing perceptions and norms by specifying exposure as well as access and price, which influence general availability.^24^,^25^ Therefore, when big changes in policies targeting one substance occur, such as those recently directed toward cannabis, they provide a useful opportunity for identifying the true relationship between the demand for the targeted substance and any related substance, in this case alcohol. However, as Figure 1 shows, the opportunity to identify the nature of the relationship between cannabis and alcohol using this sort of large change in policy toward cannabis requires two things: (1) clear evidence that the policy in question changes the use of cannabis, the substance it actually is targeting, and (2) accounting for variation in any other alcohol policy that also might be changing and influencing consumption of alcohol (and possibly cannabis) at the same time.

Several studies published in economics journals suggest that evidence from state policies supports the conclusion that alcohol and cannabis are economic substitutes.^26^–^28^ Yet, findings in the broader public health and sociology literature have been unable to draw such a firm conclusion.^21^,^29^–^31^ The difference may be due to how economists strictly define substitutes and complements, using information gleaned from cross-price effects and their impacts on the budget constraint, or it may be due to methods relied on for causal inference.

This study updates and extends the 2016 review by Guttmannova et al., which summarized the findings regarding substitution and complementary use of alcohol and cannabis from published literature through 2015.^31^ However, unlike the prior review, this article applies specific methodological standards associated with supporting causal inference^32^,^33^ in screening the studies reviewed, with the goal of generating a more consistent interpretation of the literature evaluating the impact of cannabis policy on alcohol use and co-use with cannabis. This review discusses differences in effects identified across age groups, measures of cannabis policy (medical marijuana laws or recreational marijuana laws), and polydrug use (simultaneous and/or concurrent use).

By focusing on studies that generate findings from policy variation, this paper excludes studies such as those conducted by Park et al.^34^ and O’Hara et al.,^35^ which also examined the relationship between alcohol and cannabis use, but without considering the role policies play by influencing the relative access and price of each substance. Economists rely on changes in patterns of use associated with exogenous shifts in prices, particularly the full price of related goods, for identification of economic substitutes or complements. Legal policy changes influence the monetary and legal cost of accessing a substance, and hence they are considered components of the full price of a substance.^24^ However, as shown in this review, many studies, including those within the economics literature, have relied on a relatively weak measure of state alcohol policy, the beer-specific excise tax. Prior work has shown that over the past 20 years, the beer-specific excise tax accounts for a small percentage of taxes and is a poor indicator of alcohol taxes compared to measures incorporating multiple tax and beverage types.^36^,^37^ Exclusion of the many additional dimensions of alcohol policy measures that influence the alcohol policy environment and the full price of alcohol may lead to an omitted variable bias when examining the impact of changes in cannabis policy. Thus, a key contribution of this literature review is its consideration of the extent to which studies have appropriately considered the true availability of alcohol while interpreting findings related to cannabis policy.

## Material and Methods

### Search Strategy

The authors followed many of the PRISMA 2020 Guidelines for conducting and reporting the findings from this literature review.^38^ An online literature search was conducted for articles published between January 1, 2015, and December 31, 2020, using the following databases: EBSCO (which includes Academic Search Complete, American Psychological Association (APA) PsycInfo, Criminal Justice Abstracts, EconLit, Index to Legal Periodicals & Books, National Criminal Justice Reference Service Abstracts, and Social Sciences Abstracts), APA PsycArticles, PubMed, Scopus, Sociological Abstracts, and Web of Science. Additional search limiters were imposed related to language (English only) and study setting (United States and Canada only); and nonhuman studies, conference abstracts, and dissertations were explicitly excluded. The search terms used closely follow those of Guttmannova et al.,^31^ with two important differences. First, some additional search terms were included to capture more inclusively cannabis and alcohol use behaviors (e.g., the terms “cannabis,” “beer,” “wine,” and “spirit” had all been excluded from the Guttmannova study^31^ but were included in this study). Second, this study excluded the requirement that the paper explicitly include one of the terms “spillover/complement*/substit*” to identify papers where information could be gleaned about this relationship even if it was not the primary purpose of the study. The final search term algorithm included (marijuana/marihuana/cannab*/pot/weed/THC) and (medical/nonmedical/recreat*/“adult-use”/decrim*/policy/ policies/liberal*/law/legal*) and (alcohol/drink*/beer/ethanol/etoh/wine/spirit*/liquor).

### Inclusion Criteria

Two senior researchers independently screened all titles and abstracts to identify articles for exclusion because they were reviews, commentaries, or descriptive in nature, or because they did not include an outcome clearly identified as related to alcohol. Studies deemed eligible by at least one reviewer were included for full-text review and assigned to one of the authors of this report to read, assess for methodological strengths of the study, and extract data for coding of studies. At this stage, additional articles were excluded if (1) the study did not examine the effect of a change in cannabis policy on an alcohol-specific outcome, which is the same criteria used by Guttmannova et al.;^31^ or (2) the study did not use a methodologically appropriate design for the identification of plausibly causal policy impacts on the alcohol-related outcome. Methodological designs deemed inappropriate for identification of policy effects were those that either had no within-state or out-of-state control group or did not use a pre-post analytic design.

### Data Extraction

A standardized Excel form was used to extract information by each reviewer on the details of the reviewed papers, including the study’s data source(s); years covered; policy measures and sources; population included; methods used; specific alcohol-related, cannabis-related, and other outcome measures examined; statistical significance and magnitude of findings; and study limitations.

### Quality Assessment

Although inclusion criteria restricted the sample to studies that are methodologically strong in terms of use of a comparison or control group and use of pre- and post-policy evaluation design, additional aspects of these studies are important for considering the reliability of the findings. First, consideration of the data set used for identification of findings is important as studies with state-representative samples—such as the National Survey on Drug Use and Health,^39^ the state-specific Youth Risk Behavior Surveillance System,^40^ and several state-specific school surveys—produce more reliable findings than data sources that do not consist of state-representative samples for all states, such as the Monitoring the Future^41^ survey.^42^,^43^ Second, the time period in which policies are evaluated is important as it can influence which states provide variation to identify policy effects, and states that adopted policies prior to 2010 were far more lenient in terms of market regulation than were states that have adopted policies since then.^44^ Third, the specific cannabis policy being evaluated might matter, as well as the specific policy dimensions, as these policies may influence use through different mechanisms, including norms, availability/access, and/or price.^25^ Fourth, as mentioned already, many studies fail to include a measure for alcohol policy over the same time period or may include what the literature has deemed a relatively weak measure of the overall alcohol policy environment, which might generate omitted variable bias in the analysis.^36^ Finally, the authors considered the reliability of the finding not just in terms of the significance and directionality of findings for alcohol but in terms of cannabis as well. For example, a policy that was associated with a significant decrease in alcohol use without a corresponding significant increase in cannabis use seems unlikely to truly reflect substitution between the two substances. All of these aspects were considered when assessing the actual findings from each study.

### Search Results

**Figure 2:**
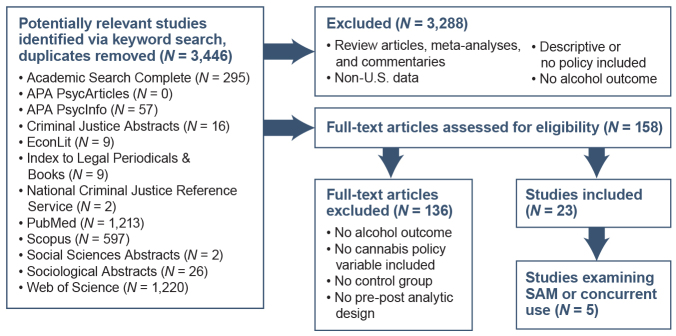
Flow diagram showing search algorithm results and inclusion/exclusion process to generate final studies included in this review. *Note:* APA, American Psychological Association; SAM, simultaneous use of alcohol and marijuana.

The search identified 3,446 unique monographs (Figure 2). Title and abstract screening led to the exclusion of the majority of the identified articles (*N* = 3,288). The remaining 158 articles underwent full-text assessment, from which 23 were included in this review.

## Results

Table 1 summarizes key characteristics of the 23 included studies. Study time frames span from 1977 to 2018, with study periods ranging from 4 to 39 years, and most studies (*n* = 15) included data from all or most U.S. states. Medical marijuana laws (MMLs) were the most common policy of interest (16 studies), followed by recreational marijuana laws (RMLs) (11 studies) and decriminalization (four studies). Seven of the 16 MML studies evaluated specific legal provisions (e.g., allowance for home cultivation) or implementation features (e.g., dispensaries operational); specific provisions of decriminalization and RMLs were not assessed, with the exception of the study by Hansen et al.,^28^ who defined RML policy timing based on retail store availability. Regarding alcohol-related outcomes, most studies evaluated cannabis policy effects on self-reported prevalence or frequency of use (*n* = 10) or heavy or binge drinking behavior (variously defined across studies; *n* = 8), with relatively fewer studies evaluating alcohol-related driving or traffic fatality outcomes (*n* = 5), health care service utilization (*n* = 3), or sales data (*n* = 2).

Seven studies provided estimates specific to youth populations (generally adolescents no older than high school seniors), and six provided estimates for young adults (generally those who have entered college or are of college age, between the ages of 18 and 29). Many studies focusing on the impact of cannabis policies on cannabis use have found differing effects of these policies by age group; youth prevalence rates have generally been found to be insensitive to cannabis policies,^44^,^45^ whereas prevalence rates in young adult and adult populations have generally been found to be positively associated with these cannabis liberalization laws.^18^,^44^ This review’s findings on the impact of cannabis policy on alcohol use across studies are reported by age group.

### Cannabis Policies and Alcohol Use by Youth and Young Adults

Table 2 summarizes findings on the impact of medical and recreational cannabis policies on alcohol use as well as the key characteristics of the 13 included studies that assessed youth and/or young adult populations. Outcomes for measures of alcohol and cannabis use are reported in terms of direction and statistical significance in the final two columns. Those results that meet the standard threshold of statistical significance (alpha = 0.05 for a two-tailed test) are shown in bold.

Among the youth and young adult populations studied, findings regarding measures of use were inconsistent across data sets and studies, with some studies showing an increase in 30-day alcohol use with medical cannabis laws^46^,^47^ and others showing a decrease.^48^,^49^ Similarly, some studies noted an increase in binge drinking^48^ whereas others detected a decrease in binge drinking.^49^,^50^

#### Findings for youth

Before interpreting these mixed results for youth by considering several factors (e.g., the measure of cannabis policy, years being evaluated, data sets used, inclusion of other alcohol policy measures), it is important to first note that the findings of the impact of these same policies on cannabis use from the same study (e.g., same population, same measure, same time period) were similarly inconclusive. Most of the studies showed that the impact of cannabis policies on cannabis use was statistically insignificant for youth, with few exceptions: Wen et al. suggested that cannabis liberalization was negatively associated with age at first use (i.e., more liberal policies were associated with earlier initiation), and that retail dispensaries specifically increased past-month use.^48^ Cerdá et al. determined that MMLs were negatively associated with marijuana use among 8th graders only,^50^ and Bailey et al. suggested that RMLs were positively associated with cannabis use in the past year.^51^ These exceptions do not tell a consistent story of the impact of cannabis policy on cannabis use among youth and reinforce conclusions from earlier literature reviews.^25^

Given the inconsistency in cannabis policy effects on measures of cannabis use among youth, and that most of these studies detected no significant impact of the policy on cannabis use at all, it makes sense to focus on studies determining that a given measure influences cannabis use before trying to infer the measure’s impact on alcohol use. A couple of studies showed significant impacts of cannabis policy on alcohol use—consistently suggesting that liberalization of these policies reduced alcohol use by youth.^49^,^52^ In these studies, however, the policy was not significantly associated with cannabis use, with the exception of the study by Johnson et al., where the association was negative.^49^ Only Bailey et al. found evidence of statistically significant increases in both past-year cannabis use and alcohol use,^51^ with these results suggesting that cannabis and alcohol are economic complements. However, this study did not control for alcohol policy, raising concerns that this finding may be a function of an omitted variable bias.

Findings specific to RML, which is enacted only in states that have already passed MML, were also inconsistent. Coley et al. found a decrease in past-month cannabis use and level of alcohol use,^46^ whereas Mason et al. detected an increase in cannabis use and a decrease in alcohol use,^52^ and Bailey et al. showed increases in both cannabis and alcohol use.^51^ The differences in findings are likely a function of the time periods examined, controls being included, and states sampled. Mason et al.^52^ and Bailey et al.^51^ similarly focused only on RML policies in just a few states but evaluated very different pre-period trends, while Coley et al.^46^ covered a long time period similar to that of Bailey et al.^51^ but also considered the influence of adopting MML and decriminalization statutes as well as included data from all 50 states.

Alcohol policies, with the exception of beer taxes, generally were ignored in the youth-focused studies in Table 2. In the one study that included a broader set of alcohol policies in the mix, the findings regarding alcohol and cannabis use were statistically insignificant.^47^

The fact that these seven well-designed studies generated inconsistent and generally insignificant results regarding the impact of cannabis policy on alcohol (as well as cannabis) use, leads the authors to conclude that the current state of the science regarding the impact of cannabis policy on youth cannabis and alcohol use is inconclusive. This is not to say that there is no relationship between cannabis policy and alcohol use, however, as the authors do not believe there are enough scientifically robust studies to draw such a conclusion using state-representative samples (i.e., National Survey on Drug Use and Health^39^ and Youth Risk Behavior Surveillance System^40^) with strong alcohol policy controls included.

#### Findings for young adults

Table 2 also includes results from six studies that specifically assessed young adult populations. Across these studies a bit more consistency exists in that all studies in this group showed a negative association between RML/MML and past-month drinking and/or binge drinking. At least one study also showed a negative association between MML and alcohol-involved motor vehicle crashes (for accidents involving at least one individual with a blood alcohol concentration greater than 0.08%).^53^ However, only two of the studies looked at the effects of cannabis policies on cannabis use within the population directly. One of the studies yielded a positive association between RML and cannabis use,^54^ and the other study yielded a statistically insignificant result.^55^ If cannabis liberalization policies do not directly influence cannabis use measures among the young adult population, it calls into question any causal association between liberalized cannabis policies and reduced alcohol use measures, at least with respect to a substitution hypothesis.

Two studies examining RML specifically showed a negative association between cannabis liberalization policy and heavy or binge drinking.^55^,^56^ Only the study with an insignificant association provided evidence supporting a potentially causal relationship due to direct effects on cannabis, but it examined regular alcohol use and did not include any controls for alcohol policies.^54^ All three studies that examined the effect of MML on young adult alcohol use included some measure of alcohol policy,^26^,^53^,^57^ and two of these studies^26^,^57^ showed statistically significant impacts on drinking. However, as noted already, none of the studies showed a positive association between MML policy and cannabis use.

Thus, while the findings across studies for young adults indicate a more consistent association between cannabis liberalization policies and alcohol use (one supporting possible substitution), the authors do not believe the evidence in total supports the conclusion that alcohol and cannabis are substitutes for this age group. There remain too many limitations of the existing literature to support such a robust conclusion, particularly in light of evidence showing higher prevalence of simultaneous use.^19^

### Cannabis Liberalization Policy and Adult Alcohol Use

Table 3 reports the same information as Table 2, but focuses on the 14 included studies that reported results for the entire adult population. Given differences in the types of data assessed across these 14 studies, this paper considers the results by source of data. In other words, this review looks first at findings from studies using data from self-reported use, next examines findings from studies focusing on populations seeking care from the health system, then considers findings from studies using alcohol sales data, and finally considers results obtained from crash data.

#### Findings from self-reported use in population surveys

The authors identified two studies in this group that presented findings of the impact of cannabis policy on cannabis use as well as alcohol use, and both studies reported that more liberal cannabis policies were associated with increased past-month cannabis use and near-daily use.^48^,^58^ However, these same two studies showed completely different impacts of their cannabis policy variable on past-month binge drinking, with Wen et al. noting an increase in past-month binge drinking days,^48^ and Dragone et al. reporting a decrease in past-month binge drinking.^58^ The difference across the two studies for alcohol use, despite similar findings for cannabis use, is likely driven by a few factors, including different cannabis policies being considered (MML and RML), different time periods being examined (2004–2012 and 2010–2014), and differences in the inclusion of alcohol policy measures (beer tax and none).

Three studies used data from the Behavioral Risk Factor Surveillance System (BRFSS),^59^ albeit examining slightly different years and adult age groups.^26^,^57^,^60^ All three studies suggested that alcohol use decreased with more liberal MML laws, although none of these studies considered the direct impact of the cannabis laws on adult cannabis use. Moreover, the two BRFSS studies that included alcohol policy measures in addition to MMLs generally showed statistically insignificant results except for binge drinking among adults age 35 and older.^26^,^57^

The last study examining self-reported use measures in survey data provided no further clarity on the relationship, as none of the results were statistically significant, although the outcome measures used in this study were driving under the influence of alcohol or cannabis, not use in the past month or year.^61^

#### Findings from populations seeking health care services

Three studies included in this review focusing on the general population drew on data from different sectors of the health care system, and yet all three studies suggested that changes in cannabis policies were associated with an increase in both cannabis-involved and alcohol-related health care utilization.^62^–^64^ The time periods examined differed quite a bit across the studies. In addition, studies examined different types of health care utilization, such as emergency department visits from a single state; hospital admission data for individuals diagnosed with marijuana abuse criteria using codes from the *International Classification of Diseases 9th Revision* (*ICD-9*);^65^ and treatment admissions from the Treatment Episode Data Set^66^ that includes people meeting cannabis abuse criteria according to the fourth edition of the *Diagnostic and Statistical Manual of Mental Disorders* (*DSM-IV*),^67^ which distinguishes between substance abuse and substance dependence. The finding of statistical significance for both cannabis- and alcohol-related outcomes within the same data set in two of the three studies is reassuring for interpreting the results for the alcohol-involved outcome, although only one study included a measure of alcohol policy in their model,^62^ raising concerns again of omitted variable bias. These limitations aside, it is striking how different the suggested relationship between alcohol and cannabis (evidence of complementarity) is from these health care system data compared with the self-reported survey data (evidence suggesting substitution). The difference may be a function of the fact that those seeking health care services may represent a different, perhaps more at-risk, population than those reporting in household surveys (i.e., women who are pregnant, people at risk of an overdose, and/or those meeting *DSM-IV* criteria for alcohol or cannabis abuse).

#### Findings from sales data

Two studies included in this review focused on population aggregated sales data, either in terms of total aggregated sales of alcoholic beverages per capita^68^ or in terms of Nielsen scanner data sales.^27^ The findings from these two studies suggested that alcohol sales were lower in states that adopted MMLs. However, the findings in the study by Veligati et al., which also included additional alcohol policy measures that better captured the overall alcohol environment and covered a much longer time period, suggested that this association was not statistically significant.^68^ Moreover, Veligati et al. suggested that states that further adopted adult-use cannabis policies subsequently had an increase in per capita alcohol sales;^68^ Baggio et al. did not consider these subsequent changes.^27^

#### Findings from fatal crash statistics

Despite numerous examinations of the impact of cannabis liberalization policies on fatal alcohol-involved crashes, only three studies made it through this review screen.^28^,^53^,^69^ The three studies focused on very different age groups, cannabis policies, and time periods, so it is perhaps not surprising that here, too, no clear conclusions can be drawn. Although Cook et al. included some measures for alcohol policy and separately evaluated the impact of decriminalization and medical cannabis policies, the study did not include evidence of the direct effect of these policies on cannabis-related driving fatalities.^53^ Thus, it is unclear if the decline in motor vehicle crashes associated with more significant drinking (blood alcohol concentration ≥ 0.08%) represented a true substitution effect. Although both Steinemann et al.^69^ and Hansen et al.^28^ also considered cannabis-involved crashes, neither found a significant impact on alcohol-involved crashes. The lack of an association, however, may reflect an omitted variable bias caused by the lack of controls for alcohol policies. Only Steinemann et al. included years prior to 2000, thereby capturing impacts of the early adopting medical cannabis states (California, Oregon, and Washington).^69^ The heterogeneity in study designs makes it unwise to conclude that the inconsistent findings are evidence of no impact of these policies, but the findings also demonstrate the need for more consistent approaches across studies.

### Cannabis Policies and Simultaneous/Concurrent Use Outcomes

This paper identified only five studies, summarized in Table 4, that met the inclusion criteria and considered the impact of cannabis policy on concurrent or simultaneous use of alcohol and cannabis.^48^,^49^,^55^,^61^,^69^ None of these studies fully accounted for alcohol policies, despite including explicit measures of alcohol use. The two studies examining concurrent use among youth populations showed that concurrent use of cannabis and alcohol use/binge drinking generally both declined with adoption of medical cannabis policies,^48^,^49^ but the findings were only statistically significant in the Johnson et al. study,^49^ which did not control for the alcohol policy environment. In the one study examining young adults, Kerr et al. found that concurrent use of cannabis and heavy alcohol use increased with adoption of recreational cannabis laws,^55^ but again the study did not account for the alcohol policy environment. The remaining three studies that examined concurrent and simultaneous use for adult populations generally supported complementary findings (like those for the young adults);^48^,^61^,^69^ however, there again were inadequate controls for alcohol policy, with only Wen et al. including a measure of the beer excise tax.^48^ Given the limited number of studies and the clear methodological concern related to omitted variable bias, it would be unwise to draw a conclusion from these results.

## Discussion

Despite being more than 20 years into the U.S. states’ experiment with medical cannabis and nearly a decade into the experiment with recreational cannabis, the scientific literature remains unclear as to the impact of these liberalization policies on alcohol use. Although the number of studies has grown substantially, even since the previous comprehensive 2016 review conducted by Guttmannova et al.,^31^ there remains insufficient evidence—both in terms of quantity and quality—to conclude that cannabis policy liberalization in U.S. states is associated with either increases or decreases in alcohol use or alcohol-related outcomes. The lack of a clear or consistent association exists mainly for medical cannabis policies, whereas for recreational cannabis policies the principal issue is a relatively small number of studies meeting inclusion criteria. Regarding relationships between cannabis policies and the concurrent or simultaneous use of alcohol and cannabis, this review also found no clear indication of an association one way or another; primarily because only a small number of unique studies met the inclusion criteria. Overall, the findings in this review, although inclusive of more recent studies, are broadly consistent with earlier findings from Guttmannova et al.^31^

It is possible that the inconclusive findings are a reflection of the fact that there may not be a meaningful or detectable association between cannabis policy liberalization and alcohol use. However, as noted throughout this review, it is also possible that the inconclusive findings pertaining to MMLs may be partly related to inconsistencies in research methods. Even studies that would be considered methodologically strong by including a comparison group and pre-post policy design often excluded relevant indicators to fully capture changes in the alcohol policy environment as well as the cannabis policy environment. Studies trying to assess the impact of RMLs on alcohol use might not yet have had sufficient time to properly evaluate their effects, particularly given the lag in opening markets after laws have passed and for markets to mature. Furthermore, the complement versus substitute nature of the relationship between cannabis and alcohol might vary based on prevalence, intensity, and frequency of use, which at this point the scientific literature is too limited to assess for reasons already discussed.

Because associations were not conclusive, the authors of this review default to the null hypothesis that (at least currently) there is no meaningful relationship between cannabis liberalization policies and alcohol use outcomes. Of course, it may be that that this conclusion is due to this review’s efforts to try to pool evidence from across very different user groups, outcomes, and policies. More systematic studies considering heterogeneous effects across these dimensions will need to be considered, such as recent work by Kim et al. in 2021.^70^ As the literature expands, attention paid to consistency across these sorts of dimensions may generate different conclusions.

### Limitations

This review has several limitations. First, it is possible that cannabis policy is related to alcohol use through other mechanisms not captured by this review. The authors have focused on studies that examined an association between cannabis policy and alcohol use through a specific mechanism in which cannabis use is a mediator. However, it is possible that changes in public perception, norms, and cannabis use have led to changes in cannabis policy. Studies that focus on this mechanism, and any others, would have been excluded from this review even if they also showed an impact on alcohol use. Another limitation is that this review’s inclusion criteria required a given study to examine the link between cannabis policy and both cannabis and alcohol use. The authors recognize that researchers may present evidence of the impact of cannabis policy on cannabis use separately from the impact of cannabis policy on alcohol use. For example, although Alley et al. did not examine the impact of RML on cannabis use,^56^ a companion paper by Bae and Kerr did find a positive association between RML and cannabis use using the same data set and time period.^71^

### Recommendations for Future Research

Perhaps the greatest contribution of this work is its identification of several key limitations in the literature, which should be better addressed in future work. In particular, studies should test the presumed intermediary causal mechanism between cannabis policy change and alcohol use. Specifically, if the mechanism of a reduction in alcohol consumption due to liberalized cannabis policies is thought to be through a substitution of cannabis for alcohol use, then studies should examine changes in cannabis use as a possible mediator of relationships between cannabis policies and alcohol use outcomes. Failing that, studies should at least report the change in cannabis consumption among the study population.

This review found no studies that formally examined cannabis use as a mediator in the relationship between cannabis policies and alcohol use outcomes. Ideally, one would have large-scale individual-level longitudinal data that would allow for the estimation of such mediation requests with attention to the temporal/causal sequencing among use of the two substances. Although several individual-level longitudinal data sets measure substance use behaviors—including the Monitoring the Future survey,^41^ the National Longitudinal Survey of Youth,^72^ and most recently the Adolescent Brain Cognitive Development Study^73^—these data sets have the limitation of small sample sizes that do not support state-representative analyses, which can cause problems for evaluating state-level policies (see Dilley et al.^43^ for a discussion of these issues and inconsistency in findings regarding policy effectiveness). Given that most studies rely on repeated cross-sectional surveys (e.g., National Survey on Drug Use and Health^39^ and BRFSS^59^), future research may improve our understanding by testing whether those subgroups or subpopulations that experience greater changes in alcohol use following cannabis liberalization also experience greater changes in cannabis use. In light of these issues with existing survey data, a richer understanding of the relationship between cannabis policy and alcohol use may be developed by synthesizing evidence from the types of causal inference studies reviewed here with evidence from high-quality epidemiological studies that may have smaller sample sizes but richer person-level data on changes in substance use patterns.

Another obvious limitation of the existing literature is that nearly half of the studies included in this review failed to assess the impact of the change in cannabis policy on cannabis consumption among the study population. Among those that did, many showed no impact of cannabis policy on cannabis consumption directly. Therefore, although alcohol use measures may have decreased around the time these cannabis policies were adopted, these data do not constitute clear evidence of substitution. Future research will need to consider nuanced measures of cannabis use, as frequency of use does not accurately reflect amount consumed overall or exposure to delta-9-tetrahydrocannabinol (THC), the main psychoactive ingredient in cannabis. If liberalized cannabis policies impact the potency (i.e., THC concentration) or types of formulations or cannabinoids consumed,^74^,^75^ then studies focused on frequency will miss important changes in use.

A third limitation is that most studies do not adequately account for alcohol policies, which are strongly related to alcohol consumption. Specifically, more than half of the studies did not account for any alcohol policies, and those that did typically accounted only for the specific excise tax for beer. Taxation is but one of many policies, and even in terms of tax policies, specific excise taxes (i.e., beer) represent only a small percentage of the total price of alcohol and are smaller in magnitude than other tax types that are applied to alcohol. Other policies affecting alcohol availability, such as outlet density or hours of sale, also may be important to include. Additionally, it may be important to consider social determinants of alcohol consumption, such as liberal state politics or religious affiliation that are statistically associated with political and/or social movements favoring the liberalization of cannabis policies that could result in associations between cannabis policies and alcohol consumption-related outcomes.

A fourth limitation is the treatment of RML and MML policies as monolithic across states, rarely examining state-level variations in policies, evaluating policy components or the timing of policies. It is not a trivial point to note that every state that adopted an RML policy prior to 2020 transitioned from a MML policy. It is possible that positive associations that were identified for RML policies in hospital admissions data^63^ and/or aggregated sales data^68^ may reflect the longer term impact of a mature market on the medical and recreational cannabis markets combined rather than an isolated impact of RML alone. Relatedly, they also might reflect a changing cannabis policy environment due to differential implementation caused by a specific federal response and/or changes in implementation that occur over time. Studies also may vary in their treatment of policy timing—whether the date for RML or MML corresponds to the date of passage, enactment, or implementation of the law (e.g., first day of retail sales), which may influence whether or not the policy is shown to have an impact on cannabis or alcohol use. Additionally, variation within states—for example, across municipalities that do or do not permit cannabis sales regardless of statewide policy—or differences in retail availability provide another opportunity for future research.

Future research will need to consider how the evolving cannabis state markets and federal position lead to changes in how a given law is interpreted by market participants, which will influence consumption of cannabis as well as any economic complement or substitute.

It is clear from the research evidence to date that the answer to the critical public health question regarding the impact of cannabis liberalization policies on alcohol use, particularly heavy drinking and drinking-related harm, remains unknown. Population evidence, such as showing that the prevalence of simultaneous use of alcohol and marijuana is increasing among those who consume high quantities of alcohol,^19^ runs counter to conclusions often drawn from a few studies that alcohol and cannabis are economic substitutes. Like the previous comprehensive review published by Guttmannova et al.,^31^ this review is unable to provide a singular interpretation of the scientific evidence to date, despite examining the more recent evidence, which has grown rapidly in the last 5 years. Significant methodological shortcomings need to be overcome before there is a clear answer to the nature of the relationship, and researchers will need to pay close attention as to whether the short-term response differs from the long-term relationship.

## Figures and Tables

**Figure 1:**
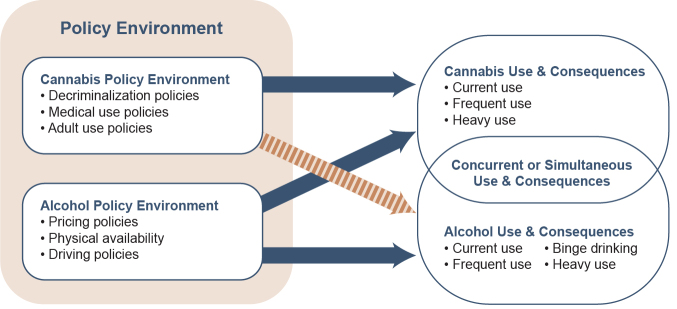
Relationship between cannabis and alcohol policy and use. Arrows represent existing relationships, with the striped orange arrow representing the relationship addressed in this review (i.e., the effects of cannabis policies on alcohol use as well as simultaneous cannabis and alcohol use and their consequences).

**Table 1:** Summary of Characteristics of 23 Included Studies

Article	Study Period	Analytic Unit	Population and Age Group(s)	MJ Policy Measure(s)	Setting	Alcohol Outcomes
Wen et al. (2015)48	2004–2012	Individual	Youth (ages 12–20) Adults (age 21+)	MML*	United States (all states)	30 d alcohol use Heavy or binge use AUD CAM use SAM use
Mason et al. (2016)^52^	2010–2013	Individual	Youth (grades 8–9)	RML	Washington State	Initiation 30 d alcohol use
Dills et al. (2017)^47^	1977–2015	Individual	Youth (grade 12)	Decrim MML/RML	United States (48 states)	30 d alcohol use Lifetime use DUIA
Kerr et al. (2017)^55^	2012–2016	Individual	College students (ages 18–26)	RML	Oregon, compared to unspecified number of states	Heavy or binge use CAM use
Sabia et al. (2017)^26^	1990–2012	Individual	All (age 18+) and some age-specific brackets	Decrim MML	United States (all states)	30 d alcohol use Heavy or binge use
Cerdá et al. (2018)^50^	1991–2015	Individual	Youth (grades 8, 10, and 12)	MML	United States (48 states)	Heavy or binge use
Johnson et al. (2018)^49^	1991–2011	Individual	Youth (grades 9–12)	MML*	United States (45 states)	30 d alcohol use Heavy or binge use CAM use
Kerr et al. (2018)^54^	2008–2016	Individual	College students (ages 18–26)	RML	Oregon, compared to non-RML states	30 d alcohol use
Sabia & Nguyen (2018)^57^	1990–2014	Individual	Adults (ages 18–64) and some age-specific brackets	Decrim MML*	United States (all states)	30 d alcohol use
Steinemann et al. (2018)^69^	1993–2015	State	All	MML	Hawaii	Crash fatalities with alcohol
Andreyeva & Ukert (2019)^60^	1993–2013	Individual	Adults (ages 18+)	MML*	United States (all states)	Heavy or binge use
Delling et al. (2019)^63^	2010–2014	State	All	RML	Colorado vs. New York & Oklahoma	Hospitalizations
Dragone et al. (2019)^58^	2010–2014	County/MSA; 3-year averages	All (age 12+)	MML RML	Washington vs. Oregon	Any use Heavy or binge use
Meinhofer et al. (2019)^62^	2002–2014	State	Pregnant women (ages 12–49)	MML	United States (48 states)	Treatment admissions
Alley et al. (2020)^56^	2008–2018	Individual	College students (ages 18–26)	RML	United States (48 states)	Heavy or binge use
Baggio et al. (2020)^27^	2006–2015	County	All	Decrim MML* RML	United States (48 states)	Sales
Bailey et al. (2020)^51^	2002–2011, 2015–2018	Individual	Youth (under age 21)	RML	Washington & Oregon	Any use
Coley et al. (2020)^46^	1999–2017	Individual	Youth (mostly ages 14–18)	Decrim MML RML	United States (47 states)	30 d alcohol use
Conyers & Ayres (2020)^64^	2010–2016	5-digit ZIP code	All	MML*	Arizona	ED visits
Cook et al. (2020)^53^	2010–2017	City	All (age 15+); ages 15–24; ages 25–44	Decrim MML	United States (large cities without MML or decrim by 2010)	Alcohol-involved crash fatalities
Fink et al. (2020)^61^	1991–1992, 2001–2002, 2012–2013	Individual	Adults (age 18+)	MML	United States (39 states)	DUIA DUIAM
Hansen et al. (2020)^28^	2000–2016	State	All (age 16+)	RML*	Washington and Colorado, compared to non-RML states	Crash fatalities
Veligati et al. (2020)^68^	1990–2016	State	All (age 14+)	MML* RML	United States (50 states)	Sales

* Indicates if estimates were obtained for specific provisions of the laws (e.g., allowance for home cultivation) or implementation aspects (e.g., retail stores open).

*Note:* AUD, alcohol use disorder; CAM, concurrent alcohol and marijuana; Decrim, decriminalization; DUIA, driving under the influence of alcohol; DUIAM, driving under the influence of alcohol and marijuana; ED, emergency department; MJ, marijuana; MML, medical marijuana law; MSA, metropolitan statistical area; RML, recreational marijuana law; SAM, simultaneous alcohol and marijuana.

**Table 2:** Summary of Findings on Impact of Cannabis Policy on Alcohol Use Measures Among Youth and Young Adults

Article	Data (Study Period)	Age Group	MJ Policy Measure	Alcohol Policy or Price Measure	Impact of MJ Policy on Alcohol Use Measure	Impact of MJ Policy on MJ Use Measure
**Youth Population (Age 19 and Younger)**						
Wen et al. (2015)^48^	NSDUH (2004–2012)	Ages 12–20	MML	Beer tax	↓ 30 d total alcohol drinks ↑ 30 d binge drinking days ↓ AUD past year	↓ 30 d MJ use ↓ 30 d daily/near daily MJ use ↓ Days of MJ use ↑ **First MJ use last year** ↓ *DSM-IV* MJ use/dependence
			MML provisions	Beer tax	NS	Retail dispensary: ↑ **30 d MJ use**
Cerdá et al. (2018)^50^	MTF (1991–2015)	Grades 8, 10, and 12	MML	Beer tax	↓ **Binge drinking, 8th graders** ↑ Binge drinking, 10th graders ↑ Binge drinking, 12th graders	↓ **30 d MJ use for 8th graders** ↑ 30 d MJ use for 10th graders ↓ 30 d MJ use for 12th graders
Dills et al. (2017)^47^	MTF (1977–2015)	Grade 12	Either MML or RML	Beer tax, MLDA, Zero tolerance law, 0.08 BAC limit	↓ Lifetime alcohol use ↓ 30 d alcohol use ↓ 30 d number of times used	↓ Lifetime MJ use ↓ 30 d MJ use ↓ 30 d number of times used
			Either MML, RML, or Decrim	Beer tax, MLDA, Zero tolerance law, 0.08 BAC limit	↑ Lifetime alcohol use ↑ 30 d alcohol use ↓ 30 d number of times used	↑ Lifetime MJ use ↑ 30 d MJ use ↑ 30 d number of times used
Johnson et al. (2018)^49^	YRBSS (1991–2011)	Grades 9–12	MML	None	↓ **30 d alcohol use** ↓ 30 d alcohol use without MJ ↓ 30 d binge drinking	↓ 30 d MJ use without alcohol
			MML Restrictiveness (Scale: Less restrictive law means higher value)	None	↓ **30 d alcohol use** ↓ **30 d alcohol use without MJ** ↓ **30 d binge drinking**	↓ 30 d MJ use without alcohol
Coley et al. (2020)^46^	YRBSS (1999–2017)	Ages 14–18+	RML	Beer tax	↓ Any 30 d alcohol use ↓ Level of 30 d alcohol use	↑ Any 30 d MJ use ↓ **Level of 30 d MJ use**
			MML	Beer tax	↑ Any 30 d alcohol use ↓ Level of 30 d alcohol use	↑ Any 30 d MJ use ↓ Level of 30 d MJ use
			Decrim	Beer tax	↑ Any 30 d alcohol use ↓ Level of 30 d alcohol use	↑ Any 30 d MJ use ↓ Level of 30 d MJ use
Mason et al. (2016)^52^	Common Sense Parenting Intervention (2010–2013)	Grades 8–9	RML	None	↓ **30 d alcohol use**	↑ 30 d MJ use
Bailey et al. (2020)^51^	Seattle Social Development Project (2002–2011–2015–2018**)**	**≤** age 20	RML	None	↑ **Past-year alcohol use**	↑ **Past-year MJ use**
**Young Adult Population (Ages 18–25)**						
Kerr et al. (2017)^55^	Healthy Minds Study (2012–2016)	Ages 18–26	RML	No	↓ Past 2-week heavy alcohol use	↑ 30 d MJ use
Cook et al. (2020)^53^	FARS (2010–2017)	Ages 15–24	DML	Beer tax ALR laws	NS: MVCs with BAC ≥ 0.08%	None
			MML	Beer tax ALR laws	↓ MVCs with BAC ≥ 0.08%	None
Alley et al. (2020)^56^	National College Health Assessment (2008–2018)	Ages 18–26	RML	No	↓ Binge drinking ≤ 20 years ↓ **Binge drinking ≥ 21 years**	None
Sabia et al. (2017)^26^	BRFSS (1993–2012)	Ages 18–24	MML	Alcohol tax Zero tolerance laws	↓ **30 d any alcohol use** ↓ **30 d binge drinking**	None
Sabia & Nguyen (2018)^57^	BRFSS (1990–2016)	Ages 18–19 Ages 20–29	MML	Beer tax	↓ 30 d number of drinks, ages 18–19 ↓ **30 d number of drinks, ages 20–29**	None
Kerr et al. (2018)^54^	National College Health Assessment II (2008–2016)	Ages 18–26	RML	No	↓ 30 d alcohol use	↑ **30 d MJ use** ↑ **Level of 30 d MJ use**

**Bold text** indicates finding statistically significant at alpha = 0.05 for a two-tailed test.

*Note:* ALR, administrative license revocation; AUD, alcohol use disorder; BAC, blood alcohol concentration; BRFSS, Behavioral Risk Factor Surveillance System; Decrim, decriminalization; DML, decriminalization marijuana law; *DSM-IV*, *Diagnostic and Statistical Manual of Mental Disorders, Fourth Edition*; FARS, Fatality Analysis Reporting System; MJ, marijuana; MLDA, minimum legal drinking age; MML, medical marijuana law; MTF, Monitoring the Future; MVCs, motor vehicle crashes; NS, nonsignificant; NSDUH, National Survey on Drug Use and Health; RML, recreational marijuana law; YRBSS, Youth Risk Behavior Surveillance System.

**Table 3:** Summary of Findings on Impact of Cannabis Policy on Alcohol Use Measures Among Studies Examining Adult Populations or All Ages

Article	Data (Study Period)	Age Group	MJ Policy Measure	Alcohol Policy or Price Measure	Impact of MJ Policy on Alcohol Use Measure	Impact of MJ Policy on MJ Use Measure
Wen et al. (2015)^48^	NSDUH (2004–2012) Individual	Age 21+	MML	Beer tax	↑ 30 d total alcohol drinks ↑ **30 d binge drinking days** ↑ AUD in past year	↑ **30 d MJ use** ↑ **30 d daily/near-daily MJ use** ↑ Days of MJ use ↑ First MJ use last year ↑ *DSM-IV* MJ use/dependence
			MML provisions	Beer tax	Nonspecific pain provision: ↑ **30 d binge drinking days**	Nonspecific pain provision: ↑ **30 d MJ use** ↑ **Daily/near-daily MJ use**
Dragone et al. (2019)^58^	NSDUH (2010–2014) Aggregated	Adult	RML	No	↓ Alcohol use ↓ **30 d binge drinking**	↑ **MJ use**
Sabia et al. (2017)^26^	BRFSS (1990–2012)	Age 25+	MML	Alcohol tax Zero tolerance laws	↓ **30 d any alcohol use, ages 25–34** ↓ 30 d any alcohol use, age 35+ ↓ Binge drinking, ages 25–34 ↓ **Binge drinking, age 35+**	None
Sabia & Nguyen (2018)^57^	BRFSS (1990–2016)	Ages 30–39 Ages 40–49 Ages 50–64	MML	Beer tax	↓ 30 d number of drinks, ages 30–39 ↓ 30 d number of drinks, ages 40–49 ↓ 30 d number of drinks, ages 50–64	None
Andreyeva & Ukert (2019)^60^	BRFSS (1993–2013)	Age 18+	MML	No	↓ **30 d heavy drinking** ↓ **30 d risky drinking** ↓ 30 d binge drinking	None
			MML with active dispensaries	No	↑ 30 d heavy drinking ↓ 30 d risky drinking ↓ 30 d binge drinking	None
Fink et al. (2020)^61^	NLAES (1991–1992), NESARC (2001–2002), NESARC-III (2012–2013)	Age 18+	MML	No	NS: Driving under influence of alcohol	↑ Driving under influence of MJ
Meinhofer et al. (2019)^62^	TEDS (2002–2014)	Ages 12–49	MML	Beer tax	↑ **Tx admissions of pregnant women involving alcohol** ↑ Tx admissions of nonpregnant women involving alcohol	↑ **Tx admissions of pregnant women involving MJ** ↑ Tx admissions of nonpregnant women involving MJ
Conyers & Ayres (2020)^64^	Hospital discharge data (2010–2016) Arizona only	All ages	MML	No	↑ ED visits for *ICD-9* alcohol abuse and poisoning	↑ **ED visits for** *ICD-9* **MJ abuse and poisoning**
Delling et al. (2019)^63^	Healthcare Cost and Utilization Project (2010–2014)	All ages	RML	No	↑ **Admissions for** *ICD-9* **alcohol abuse**	↑ **Admissions for** *ICD-9* **MJ abuse**
Baggio et al. (2020)^27^	Nielsen retail scanner data (2006–2015)	Age 21+	MML	Beer tax	↓ **Alcohol sales**	None
Veligati et al. (2020)^68^	Per capita sales (1990–2016)	All ages	MML	Beer tax Legal BAC limit of 0.08	↓ Per capita alcohol sales	None
			RML	Beer tax Legal BAC limit of 0.08	↑ Per capita alcohol sales	None
Cook et al. (2020)^53^	FARS (2010–2017)	Ages 25–44	Decrim	Beer tax ALR	↓ MVCs with BAC ≥ 0.08%	None
			MML	Beer tax ALR	↓ **MVCs with BAC ≥ 0.08%**	None
Steinemann et al. (2018)^69^	FARS (1993–2015)	All ages	MML	No	NS: Positive for alcohol among fatally injured drivers tested NS: Positive for alcohol among all fatally injured drivers	↑ **Positive for MJ among fatally injured drivers tested** ↑ **Positive for MJ among all fatally injured drivers**
Hansen et al. (2020)^28^	FARS (2000–2016)	Age 16+	RML	No	NS: Fraction of fatal accidents with 1+ alcohol-positive driver NS: Total alcohol-related fatalities per 1 billion VMT	NS: Fraction of fatal accidents with 1+ MJ-positive driver NS: Total MJ-related fatalities per 1 billion VMT

**Bold text** indicates finding statistically significant at alpha = 0.05 for a two-tailed test.

*Note:* ALR, administrative license revocation; AUD, alcohol use disorder; BAC, blood alcohol concentration; BRFSS, Behavioral Risk Factor Surveillance System; Decrim, decriminalization; *DSM-IV*, *Diagnostic and Statistical Manual of Mental Disorders, Fourth Edition*; ED, emergency department; FARS, Fatality Analysis Reporting System; *ICD-9*, *International Classification of Diseases: Ninth Revision*; MJ, marijuana; MML, medical marijuana law; MVCs, motor vehicle crashes; NESARC, National Epidemiologic Survey on Alcohol and Related Conditions; NLAES, National Longitudinal Alcohol Epidemiologic Survey; NS, nonsignificant; NSDUH, National Survey on Drug Use and Health; RML, recreational marijuana law; TEDS, Treatment Episode Data Set; Tx, treatment; VMT, vehicle miles traveled; YRBSS, Youth Risk Behavior Surveillance System.

**Table 4:** Summary of Findings on Impact of Cannabis Policy on Concurrent or Simultaneous Use of Alcohol and Cannabis

Article	Data (Years Analyzed)	Age Group	MJ Policy Measure	Alcohol Policy Measure	Impact on Measure of Concurrent (C) or Simultaneous (S) Drinking
**Youth Population (Age 19 and Younger)**					
Wen et al. (2015)^48^	NSDUH (2004–2012) Individual level data	Ages 12–20	MML MML provisions	Beer tax	↓ 30 d cannabis use and binge drinking (C) ↓ 30 d cannabis use while drinking (S)
Johnson et al. (2018)^49^	YRBSS (1991–2011)	Grades 9–12	MML	No	↓ **30 d alcohol and cannabis use (C)**, but no effect on 30 d cannabis use without alcohol or 30 d alcohol use without MJ
			MML restrictiveness (Scale: Less restrictive law means higher value)	No	↓ **30 d alcohol and cannabis use (C)**, but no effect on cannabis use without alcohol
**Young Adult Population (Ages 18–26)**					
Kerr et al. (2017)^55^	Healthy Minds Study/ College Students (2012–2016)	Ages 18–26	RML	No	↑ **30 d MJ use among heavy alcohol users in past 2 weeks (C)**
**Adult Population (All Ages ≥ 18)**					
Fink et al. (2020)^61^	NLAES (1991–1992), NESARC (2001–2002), NESARC-III (2012–2013)	Ages 18+	MML	No	↑ Self-reported driving under the influence of alcohol and MJ (S)
Wen et al. (2015)^48^	NSDUH (2004–2012) Individual level data	Ages 21+	MML MML provisions	Beer tax	↑ **30 d MJ use and binge drinking (C)** ↑ **30 d MJ use while drinking (S)** Nonspecific pain provision: ↑ **30 d MJ use and binge drinking (C)** ↑ **30 d MJ use while drinking (S)**
Steinemann et al. (2018)^69^	FARS (1993–2015)	All ages	MML	No	↑ **Alcohol impairment for THC-positive drivers vs. non-THC-positive drivers**

**Bold text** indicates finding statistically significant at alpha = 0.05 for a two-tailed test.

*Note:* (C), concurrent; FARS, Fatality Analysis Reporting System; MJ, marijuana; MML, medical marijuana law; NESARC, National Epidemiologic Survey on Alcohol and Related Conditions; NLAES, National Longitudinal Alcohol Epidemiologic Survey; NS, nonsignificant; NSDUH, National Survey on Drug Use and Health; RML, recreational marijuana law; (S), simultaneous; THC, delta-9-tetrahydrocannabinol; YRBSS, Youth Risk Behavior Surveillance System.
